# A Review on Phytochemistry and Pharmacology of *Cortex Periplocae*

**DOI:** 10.3390/molecules21121702

**Published:** 2016-12-10

**Authors:** Yang Li, Jin Li, Kun Zhou, Jun He, Jun Cao, Mingrui An, Yan-Xu Chang

**Affiliations:** 1 Tianjin State Key Laboratory of Modern Chinese Medicine, Tianjin University of Traditional Chinese Medicine, Tianjin 300193, China; llyangtcm@163.com (Y.L.); zishan826@126.com (J.L.); z.k.ken@263.net (K.Z.); hejun673@163.com (J.H.); 2 Tianjin Key Laboratory of Phytochemistry and Pharmaceutical Analysis, Tianjin University of Traditional Chinese Medicine, Tianjin 300193, China; 3 College of Material Chemistry and Chemical Engineering, Hangzhou Normal University, Hangzhou 310036, China; 4 Department of Surgery, University of Michigan, Ann Arbor, MI 48109, USA; mingruia@umich.edu

**Keywords:** *Cortex Periplocae*, traditional Chinese medicines, periplocin, phytochemistry, biological activities

## Abstract

*Cortex Periplocae*, as a traditional Chinese herbal medicine, has been widely used for autoimmune diseases, especially rheumatoid arthritis. Due to its potential pharmaceutical values, more studies about the biological activities of *Cortex Periplocae* have been conducted recently. Meanwhile, the adverse reaction of *Cortex Periplocae* is not a negligible problem in clinic. In this article, we reviewed a series of articles and summarized the recent studies of *Cortex Periplocae* in the areas of phytochemistry and pharmacology. More than 100 constituents have been isolated and identified from *Cortex Periplocae*, including steroids, cardiac glycosides, terpenoids, and fatty acid compounds. The crude extracts of *Cortex Periplocae* and its active compounds exhibit various biological activities, such as cardiotonic effect, anticancer action, and anti-inflammatory effect. This paper aims to provide an overall review on the bioactive ingredients, pharmacological effect, and toxicity of this plant. Furthermore, this review suggests investigating and developing new clinical usages according to the above pharmacological effects.

## 1. Introduction

*Periploca* is one of the genus in *Asclepiadaceae* family and is widely distributed in the north and tropical Africa, and East Asia [1]. *Cortex Periplocae* has a special feature and shape. It presents as a roll or a groove, while a few of them appear as irregular blocks and flakes. The root is 3–12 cm long, 12 cm in diameter, 0.2–0.4 cm thick. The outer surface has a gray-brown or yellow-brown appearance that is rough and easy to peel. The inner surface shows a yellow or yellowish-brown color, with relatively smooth, easy to break, irregular sections with a yellow and white color. It has a specific aromatic smell, with those having a rough surface, thick skin, simple round shape, thick aroma, and bitter taste being the best choice. It thrives well on dry slopes, sandy ground, or hillsides. It mainly grows in Shanxi, Shandong, Henan, and Hebei province in China [2]. *Cortex Periplocae* has a long history as Traditional Chinese Medicine (TCM) for the treatment of autoimmune diseases. It was first recorded in Shen Nong Ben Cao Jing 2000 years ago. Then, this plant was written about with different names in different classic books, such as “gangliupi” in *Kexuedeminjiancaoyao*, “chouwujia” in *Shandongzhongyao*, “shanwujiapi” in *Shanxizhongyaozhi*, and “xiangwujiapi” in *Sichuanzhongyaozhi*. Traditionally, *Cortex Periplocae* has been used to treat rheumatism and strengthen bones and tendons, and it is regarded as an effective tonic medicine [3]. Currently, more than 100 compounds have been identified from *Cortex Periplocae*, some C_21_-steroidal glycosides and terpenoids have demonstrated special immunosuppressive activity [1,4]. Besides its characteristic antirheumatic effect, it was shown to specifically treat heart failure. Research studies showed that the extracts of *Cortex Periplocae* exhibited cardiotonic effects on the isolated heart of rats and could strengthen cardiotonic function [5,6]. In addition, more studies have been focused on its antitumoral effect. Studies reported that *Cortex Periplocae* showed a good effect on tumor cell apoptosis and the repression of tumor growth of SW480 cells, TE-13 cells, SMMC-7721 cells, MCF-7 cells, BGC-823 cells, etc. [2,7,8]. However, poisoning incidences caused by using *Cortex Periplocae* have also been reported. As it is well known, periplocin is a poisonous component in *Cortex Periplocae*, and it shows a special cardiotoxic effect. Sun et al. studied the LD_50_ value of periplocin in mice and determined that it was 15.20 mg/kg by intraperitoneal injection. Studies also showed that it could obviously affect the electrocardiograms of guinea pigs, which indicated that it had a special toxicity [9]. It was also found that there was a good correlation between the content of periplocin and the acute toxicity of *Cortex Periplocae*. It implied that the content of periplocin should be limited in order to reduce the toxicity response when it was used in clinic [10].

Numerous components have been identified from this herb, and some of them have been proven to have an effective function for the treatment of different diseases for many years. Here, we summarized the available articles about the photochemistry, biological activities, and toxicity profile of *Cortex Periplocae*.

## 2. Methods

All the information about this plant has been obtained through searching journals, books, and theses and collected via libraries or electronic databases including PubMed, Elsevier, Google Scholar, and Springer. Due to few publications of this herb in English literature, the main source of information of this plant was from China national knowledge infrastructure (CNKI) and other classic Chinese herbal literature.

## 3. Phytochemistry

Many chemical constituents and active ingredients have been isolated from *Cortex Periplocae*, since the 1970s by researchers [11]. So far, more than 100 compounds have been isolated and identified from this plant, containing C_21_-steroidal glycosides, cardiac glycosides, terpenoids, volatile oils, fatty acids, etc.

### 3.1. C_21_-Steroidal Glycosides

**Figure 1:**
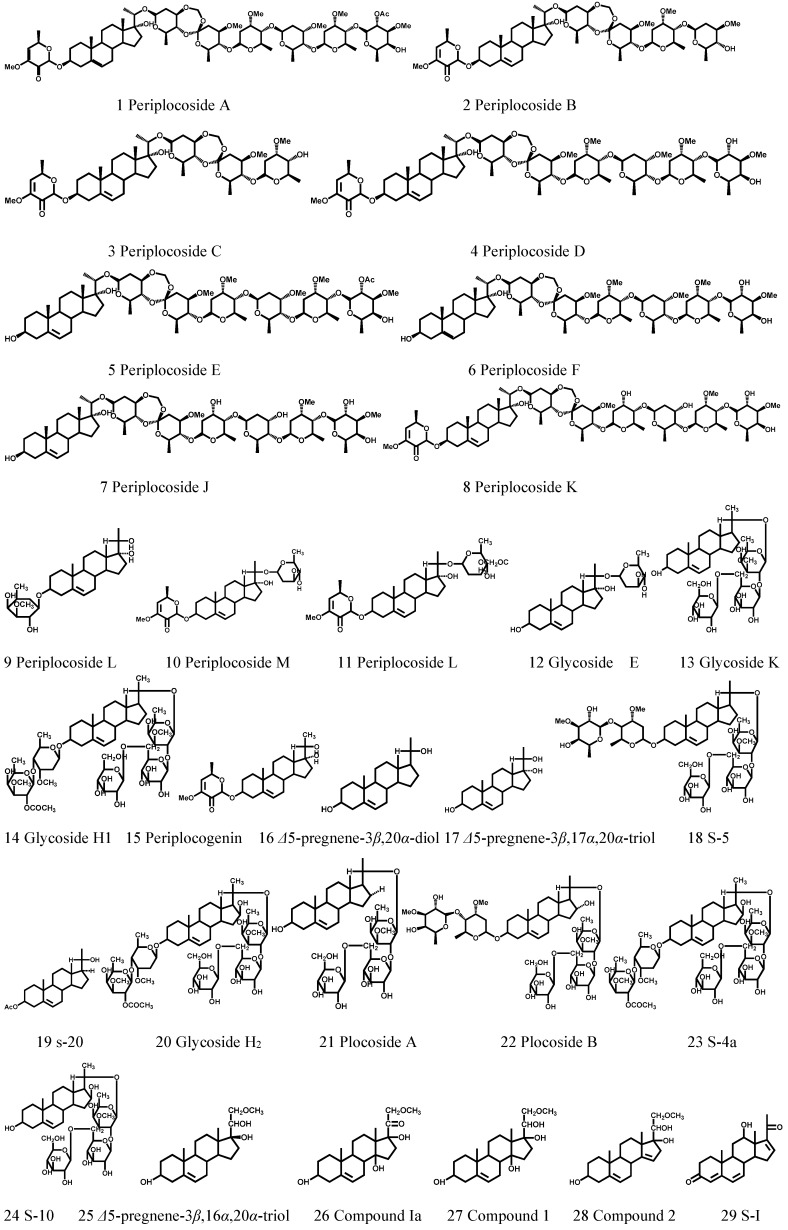

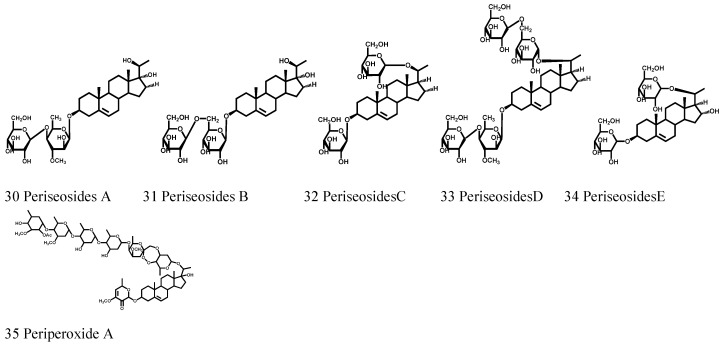
The structures of C_21_-steroidal glycosides (1–35) obtained from *Cortex Periplocae*.

C_21_-steroidal glycosides account for the majority of the total chemical constituents of *Cortex Periplocae*. There are more than 30 C_21-_steroidal glycosides which have been identified from *Cortex Periplocae* (Figure 1, Table 1). These steroids have five essential C_21_ skeletons and their respective glycosides, including β-d-glucopyranosyl (Glc), β-cymaropyranosyl (Cym), β-canaropyranosyl (Can), and β-digitalopyranosyl (Dig).

### 3.2. Cardiac Glycosides

**Figure 2:**
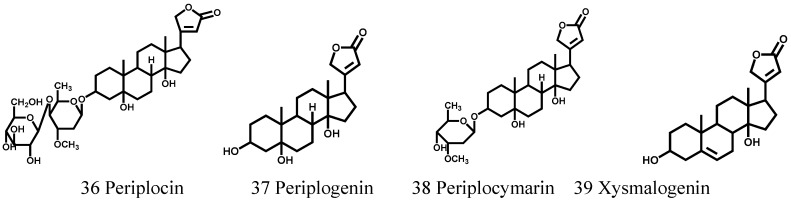
The structures of Cardiac glycosides (36–39) obtained from *Cortex Periplocae*.

Another type of compound contained in this plant is cardiac components, including two glycosides and two aglycones, which were identified as periplocin, peripocymarin, periplogenin, and xysmalogenin [23]. Research reported that periplocin was a special ingredient, which was not only a cardiotonic compound, but also the main toxic component in *Cortex Periplocae* [5]. Therefore, it is necessary to develop more studies for the assessment of toxicity of periplocin in order to guarantee efficacy and safety when it is utilized in clinic. The chemical structures of these four constituents are shown in Figure 2. Table 2 lists the compounds, plant parts used, and references.

### 3.3. Fatty Acids and Volatile Oils

**Figure 3:**
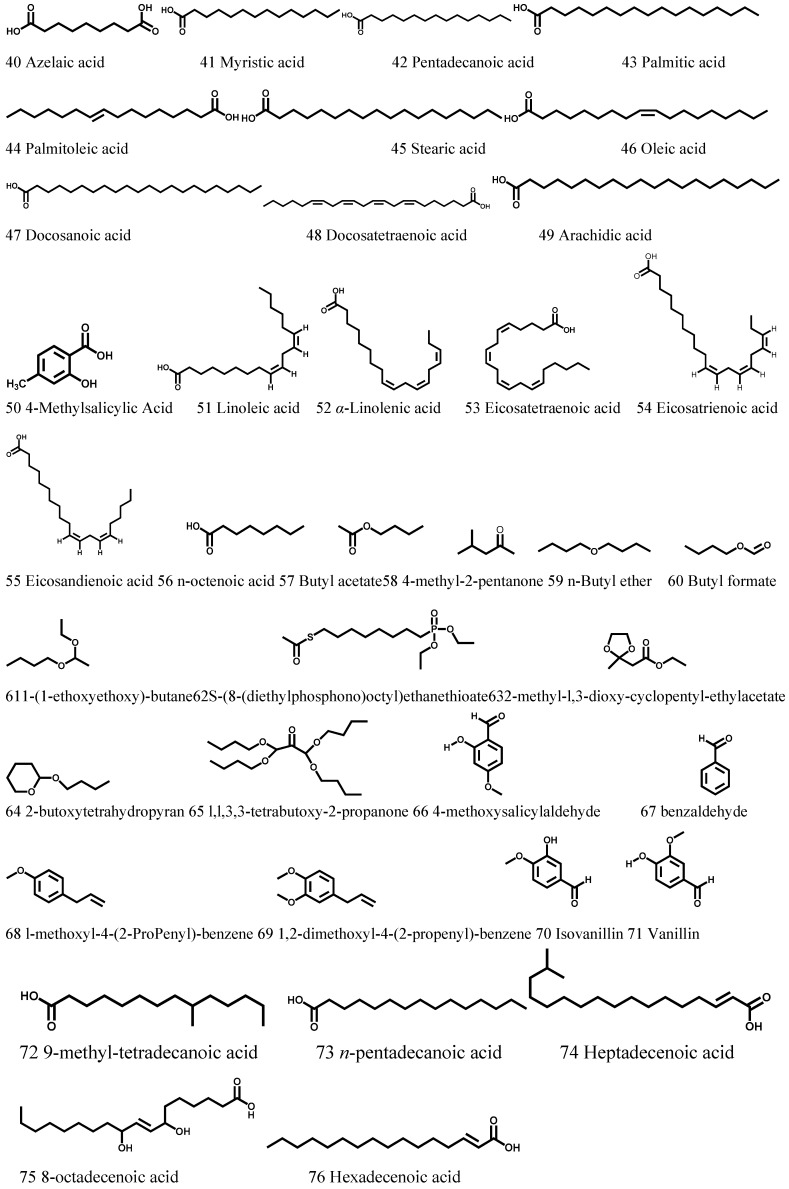
The structures of fatty acids and volatile oils (40–76) obtained from *Cortex Periplocae*.

Li et al. [25] analyzed the fatty acid compositions in the *Cortex Periplocae* extract using a gas chromatography-mass spectrometer, and identified 14 types of fatty acids (40–53), mainly including palmitic acid, linoleic acid, oleic acid, and linolenic acid. Shi et al. isolated 15 types of volatile oils (57–66) from *Cortex Periplocae* and 10 of them were purified and identified [26]. Moreover, other researchers [27] also found several different fatty acids and volatile oils in *Cortex Periplocae* (Figure 3, Table 3). 4-methoxysalicylaldehyde has a unique fragrance and expresses antimicrobial and antioxidant activities [28]. It can be deemed suitable as a standard in evaluating the quality of this plant [29].

### 3.4. Terpenoids

**Figure 4:**
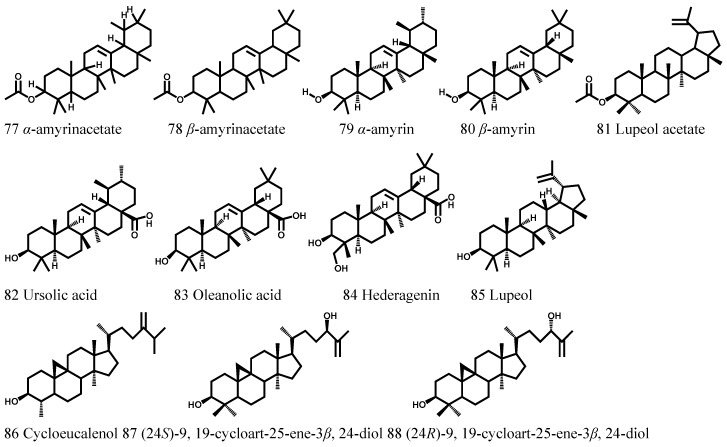
The structures of terpenoids (77–88) obtained from *Cortex Periplocae*.

Just like some other herbs, a small number of terpenoids were isolated from *Cortex Periplocae*. In 1971, Mitsuhash et al. found ursolic acid, oleanolic acid, lupeol, and lupeol acetate in *Cortex Periplocae* [31]. Furthermore, hederagenin, α-amyrinacetate, β-amyrinacetate, α-amyrin, β-amyrin, and scopoletin were identified in *Cortex Periplocae*. Later, (24*R*)-9,19-cycloart-25-ene-3β,24-diol, (24*S*)-9,19-cycloart-25-ene-3β,24-diol, and cycloeucalenol were isolated from the ethyl acetate extracts of *Cortex Periplocae* (Table 4, Figure 4).

### 3.5. Others

**Figure 5:**
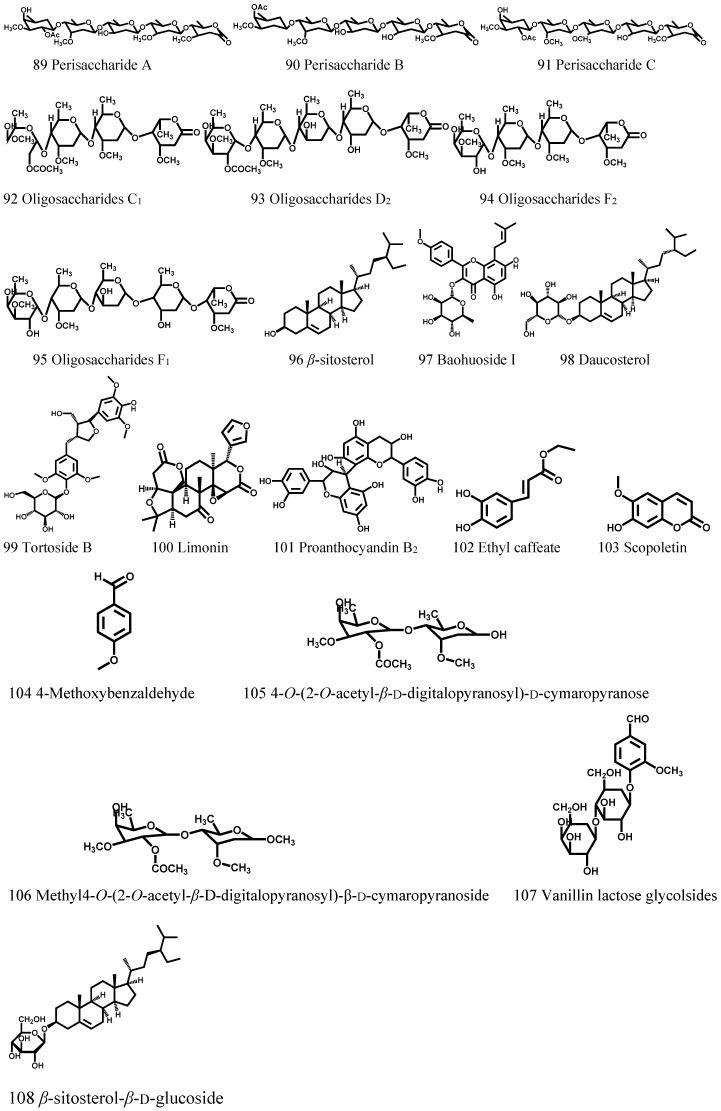
The structures of compounds (89–108) obtained from *Cortex Periplocae*.

In addition, other chemical constituents also exist in *Cortex Periplocae*, including saccharides, aldehydes, flavonoids, etc. (Table 5, Figure 5).

## 4. Pharmacokinetics

Studies on the pharmacokinetics (PK) of *Cortex Periplocae* are somewhat limited. Available literature mainly focused on its toxic component, periplocin. There is a report on the PK study of periplocin with its two metabolites (periplocymarin and periplogenin) in rat plasma and periplogenin in rat plasma. He et al. [6] studied the PK of *Cortex Periplocae* in rat’s serum. This was studied to determine the concentrations of periplocin and two metabolites simultaneously in rats’ plasma by using LC-MS/MS. The rats were given 50 mg/kg periplocin dissolved in water by gavage. As shown from the results, they found that the concentration of periplocin could only be detected at a few time points; however, its two metabolites (periplocymarin and periplogenin) reached the maximum plasma concentration after 8–10 h. The Cmax for periplocymarin and periplogenin were 1655.63 ± 404.26 ng/mL and 32.94 ± 9.16 ng/mL, respectively. Furthermore, research was done using the same method coupled with high performance liquid chromatography tandem mass spectrometry to validate the quantifying method of periplogenin in rat plasma using psoralen as an internal standard (IS) [48]. All these studies provided the pharmacokinetics (PK) information of the toxic compounds in *Cortex Periplocae*. We can better know its physiological disposition, which might be helpful for its application in clinical therapy.

## 5. Pharmacology

Recently, various studies indicate that *Cortex Periplocae* shows wide pharmacological activities, including anti-inflammatory, antitumor, cardiotonic action, nervous system action, cell differentiation, and insecticidal action, indicating that it has more potential to be discovered.

### 5.1. Anti-Inflammatory Activity

*Cortex Periplocae*, a Traditional Chinese Medicine, is widely used to treat rheumatoid arthritis in China. Based on its traditional usage, more studies are projected to find its active compounds. Tokiwa et al. verified that the aqueous extract of *Cortex Periplocae* dose-dependently inhibited the growth and IL-6 production of synovial fibroblast-like cells, which showed a good effect for cytokines [49]. Zhu et al. used bioactivity-guided isolation to screen anti-inflammatory compounds from *Cortex Periplocae* [50]. They found that periplocoside E could significantly inhibit T cell activation in vitro and in vivo. Administration of periplocoside E suppressed ovalbumin induced proliferation and cytokine (interleukin (IL)-2 and interferon (IFN)-γ) production of splenocytes in dose-dependent manners. Further studies showed that periplocoside E also inhibited anti-CD3-induced primary T cell proliferation, cytokine (IFN-γ and IL-2) production, and IL-2Rα (CD25) expression. Periplocoside A was another active compound identified from *Cortex Periplocae*. Periplocoside A could significantly suppress IL-4 transcription and IFN-γ translation, which further protected humans from autoimmune-related hepatitis [51]. These components may be responsible for the therapeutic effects of *Cortex Periplocae*. These results demonstrated that there was evidence for using *Cortex Periplocae* to treat autoimmune diseases. Furthermore, Gu et al. investigated that periplogenin could inhibit histamine release of mast cells cultured in vitro and in antigen-sensitized rats in an obvious dose-dependent manner [4]. Therefore, it was inferred that periplogenin should be one of the active compounds in *Cortex Periplocae*, producing its anti-inflammatory effects.

### 5.2. Antitumor Activity

Recently, many active screening results have indicated that *Cortex Periplocae* has antitumor activities. Du et al. used different concentrations of periplocin to determine the inhibitory effects on the proliferation of SW480 cells and observe the survival expression in SW480 cells after administering periplocin. The results showed that periplocin could significantly induce the apoptosis of SW480 cells. The SW480 cells showed some typical morphological features and microstructural changes after treating them with periplocin. Moreover, the expression of survival in SW480 cells was also inhibited after treatment with periplocin. From the results, they found the periplocin identified from *Cortex Periplocae* significantly inhibited the proliferation of SW480 cells. They further detected the expression of some signaling pathways involved in Wnt/β-catenin after treatment with periplocin, these signaling pathways in transplanted tumor were significantly lower, which was related with the inhibiting effect of the Wnt/β-catenin signaling pathway [52,53]. Zhang et al. found that periplocin inhibited the proliferation of SMMC-7721 cells and arrested them at the G2/M phase by inhibiting Stat3 signal transduction in human hepatic cells [54]. As a potential antitumor component, periplocin also showed an inhibition effect on BT-549 and TE-13, which suppressed proliferation and induced apoptosis of these cells significantly [55,56]. On the other hand, research showed that some flavonoids in *Cortex Periplocae* could inhibit the proliferation of cancer cells. For example, baohuoside I could inhibit the proliferation of Eca-109 cells in vivo and in vitro [41]. The in vitro and in vivo studies of the antitumor activity of *Cortex Periplocae* could show some potential effects to help people deal with tumors in the future.

### 5.3. Cardiotonic Action

In this plant’s traditional usages, one important application is cardiotonic action. The main compounds exhibiting cardiotonic action are cardiac glycosides. Li et al. used the isolated heart perfusion technique to evaluate the cardiotonic action of *Cortex Periplocae* [57]. The study of Li et al. indicated the *Cortex Periplocae* extracts significantly increased LVSP and decreased LVEDP, which demonstrated that *Cortex Periplocae* may have a cardiac effect. Ma et al. found periplocin could improve the structure and function of the left ventricle in chronic heart failure in rats [58]. Xie et al. proposed that cardiotonic glycosides shared the capacity to bind to the extra-cellular surface of the main ion transport protein in the cell [59]. Therefore, the mechanism of its cardiotonic action is should be explored to promote clinical administration.

### 5.4. Effects on the Nervous System

Sakuma et al. suggested that glycoside K, glycoside H1, and glycoside H2 which were identified from the *Cortex Periplocae* show strong potentiation of NGF-mediated nerve fiber outgrowth in chicken embryonic dorsal root and sympathetic ganglia, especially glycoside H2. These glycosides were consequently expected to have an impressive effect on the neuronal system [18].

### 5.5. Insecticidal Action

Zhao et al. isolated a highly potent insecticidal compound—called periplocoside NW (PSNW)—from *Cortex Periplocae* by bioactivity-guided screening method [60]. They found cell damage was caused by PSNW in the midgut of *Mythimna separata* larvae. The results of insecticidal bioassay and immunoelectron microscopic localization showed that some gold particles appeared on the microvilli layer of the midgut of *M. separata* larvae, and accumulated gradually over time until it was destroyed. Therefore, they concluded that the mechanism might be related to the degeneration of brush border microvilli, and finally periplocoside NW was applied against insects. Shi et al. found that *n*-butyl alcohol extracts from *Cortex Periplocae* could kill *Sohizaphis graminum* effectively and possessed some stomach poison against *Mythimna separata* larvae. Hui et al. separated some active components from *Periploca* methyl alcohol extraction, which exhibited significant repellent activity for *Soknopsis invicta* [61,62].

## 6. Clinic Application

### 6.1. Antirheumatic Effect

From time immemorial, *Cortex Periplocae* has been regarded as an available medicine to treat rheumatism. Because of its peculiarity, it is used as an external preparation, such as Huoxuezhitong sticking and Tianhezhuifeng sticking. Additionally, some medicinal liquors including *Cortex Periplocae* show distinct antirheumatic action.

### 6.2. Chronic Congestive Heart Failure Action

Extraction of *Cortex Periplocae* was made into tablets, 10 mg per tablet, oral administration three times a day for four or five days. In a 21 case study, 12 cases were found to be remarkable effective. It can be found that *Cortex Periplocae* has an effective action to treat chronic congestive heart failure [5].

## 7. Toxicity

Based on the traditional Chinese medicine classes, *Cortex Periplocae* is prudently recorded as a toxic medicine. *Sichuanzhongyaozhi* labeled it as “toxic, not appropriate to use for long time”. It is obvious that people long ago recognized the toxicity of *Cortex Periplocae*. Sun et al. found that all components, water extraction components, and alcohol extraction components of *Cortex Periplocae* showed the acute toxicity on mice [63]. Furthermore, through accumulative toxicity experiments, rats indicated serious reactions under a large dosage of *Cortex Periplocae* extraction [64]. Many conditions may cause intoxication such as over-dosage, drug confusion, incompatibility with herbs, and improper decoction causing life-threatening complications. Hence, it is necessary to normalize its dosage and administration.

In the Chinese Pharmacopoeia, the dosage of “xiangjiapi” ranges from 3 g/60 kg/day to 6 g/60 kg/day. Periplocin is its main toxic component, which can express cardiotonic action with unsuitable dosages. In the usual application, people always notice its great tonic effect, but not its potential toxicity. Although Tong et al. established an RP-HPLC method to determine the content of periplocin, due to extreme differences of periplocin content in *Cortex Periplocae* from different provinces, it is difficult to control toxic effects [65]. In recent years, many adverse events have often occurred. The toxic ingredients are also the effective constituents, so there is a need to strengthen knowledge about using this herb to prevent adverse outcomes. This will be beneficial in guiding reasonable clinical prescription.

## 8. Conclusions

As a traditional Chinese herbal medicine, *Cortex Periplocae* has been widely used to treat autoimmune diseases, especially rheumatoid arthritis. In the field of phytochemistry, it is reported to contain many compounds, including pregnane glycosides, steroids, volatile oils, polysaccharides, fatty acids, and cardiac glycosides. Although more than 100 compounds were obtained from this herb, further systematic phytochemistry research should be conducted. In the study of pharmacology, the extracts and chemical components from this plant focused on anti-inflammatory, antitumor, and immune functions. Although many chemical constituent analyses and pharmacological activity studies of this herb have been reported, the pharmacological mechanism of action and the metabolites responsible for these activities should be studied further. In addition, *Cortex Periplocae* was proven to be toxic in its clinical application; while its cardiotonic components are useful for the treatment of heart failure. Therefore, clarifying the mechanism, ensuring effectiveness, and minimizing risk are of great significance to more effective utilization of *Cortex Periplocae*. More studies should be carried out and methods should be developed to ensure an effective therapeutic effect with minimal toxicity in clinical applications.

## Figures and Tables

**Table 1:** C_21_-steroidal glycosides from *Cortex Periplocae* (1–35).

No.	Compound’s Name	Parts Used	Reference
1	Periplocoside A	Root	[1,12]
2	Periplocoside B	Root	[1,12]
3	Periplocoside C	Root	[1,12]
4	Periplocoside D	Root	[1,13]
5	Periplocoside E	Root	[1,13]
6	Periplocoside F	Root	[14]
7	Periplocoside J	Root	[14]
8	Periplocoside K	Root	[14]
9	Periplocoside L	Root	[1,13]
10	Periplocoside M	Root	[1,13]
11	Periplocoside O	Root	[14]
12	Glycoside E	Root	[15]
13	Glycoside K	Root	[15]
14	Glycoside H1	Root	[15]
15	Periplocogenin	Root	[1,16]
16	Δ5-pregnene-3β,20α-diol	Root	[11]
17	Δ5-pregnene-3β,17α,20α-triol	Root	[11]
18	Δ5-pregnene-3β,20(*S*)-diol 3-*O*-[β-d-digitalopyranosyl (1→4)β-d-cymaropyranoside]20-*O*-[β-d-glucopyranosyl (1→6)-β-d-glucopyranosyl (1→2)-β-d-digitalopyranoslde](*S*-5)	Root	[17]
19	*S*-20	Root	[14]
20	Glycoside H_2_	Root	[18]
21	Plocoside A	Root	[19]
22	Plocoside B	Root	[19]
23	Δ5-pregnene-3β,16β,20(*R*)-triol-3-*O*-[2,-*O*-acetyl-β-d-digitalopyranosyl (1→4)-β-d-cymaropyranoside]20-*O*-[β-d-glucopyranosyl (1→6)-β-d-glucopyranosyl (1→2)-β-d-digitalopyranoslde](*S*-4a)	Root	[17]
24	Δ5-pregnene-3β,16β,20(*R*)-trio1 20-*O*-β-d-glucopyranosyl (1→6)-β-d-glucopyranosyl (1→2)-β-d-digitalopyranoslde(*S*-10)	Root	[17]
25	Δ5-pregnene-3β,16α,20α-triol	Root	[20]
26	21-*O*-methyl-Δ5-pregnene-3β,14β,17β,21-tetraol-20-one(Compound Ia)	Root	[21]
27	21-*O*-methyl-5-pregnene-3β,14β,17β,20,21-pentaol(Compound 1)	Root	[21]
28	21-*O*-methyl-5,14-pregndlene-3β,l7β,20,21-tetraol(Compound 2)	Root	[21]
29	12β-hydroxypregna-4,6,16-triene-3,20-dione (*S*-I)	Root	[16]
30	Periseosides A	Root	[22]
31	Periseosides B	Root	[22]
32	Periseosides C	Root	[22]
33	Periseosides D	Root	[22]
34	Periseosides E	Root	[22]
35	Periperoxide A	Root	[1]

**Table 2:** Cardiac glycosides from *Cortex Periplocae* (36–39).

No.	Compound’s Name	Parts Used	Reference
36	Periplocin	Root	[24]
37	Periplogenin	Root	[1]
38	Periplocymarin	Root	[1]
39	Xysmalogenin	Root	[11]

**Table 3:** Fatty acids and volatile oils from *Cortex Periplocae* (40–76).

No.	Compound’s Name	Parts Used	Reference
40	Azelaicacid	Root	[25]
41	Myristic acid	Root	[25]
42	Pentadecanoicacid	Root	[25]
43	Palmitic acid	Root	[25]
44	Palmitoleic acid	Root	[25]
45	Stearic acid	Root	[25]
46	Oleicacid	Root	[25]
47	Docosanoicacid	Root	[25]
48	Docosatetraenoic acid	Root	[25]
49	Arachidic acid	Root	[25]
50	4-Methylsalicylic acid	Root	[25]
51	Linoleicacid	Root	[25]
52	α-Linolenic acid	Root	[25]
53	Eicosatetraenoic acid	Root	[25]
54	Eicosatrienoic acid	Root	[27]
55	Eicosandienoic acid	Root	[27]
56	*n*-octenoic acid	Root	[27]
57	Butyl acetate	Root	[26]
58	4-methyl-2-pentanone	Root	[26]
59	*n*-Butyl ether	Root	[26]
60	Butyl formate	Root	[26]
61	1-(1-ethoxyethoxy)-butane	Root	[26]
62	*S*-(8-(diethylphosphono)octyl)ethanethioate	Root	[26]
63	2-methyl-1,3-dioxy-cyclopentyl-ethyl acetate	Root	[26]
64	2-butoxytetrahydropyran	Root	[26]
65	1,1,3,3-tetrabutoxy-2-propanone	Root	[26]
66	4-methoxysalicylaldehyde	Root	[26]
67	Benzaldehyde	Root	[27]
68	1-methoxyl-4-(2-ProPenyl)-benzene	Root	[27]
69	1,2-dimethoxyl-4-(2-propenyl)-benzene	Root	[27]
70	Isovanillin	Root	[27]
71	Vanillin	Root	[30]
72	9-methyl-tetradecanoic acid	Root	[27]
73	*n*-pentadecanoic acid	Root	[27]
74	Heptadecenoic acid	Root	[27]
75	8-octadecenoic acid	Root	[27]
76	Hexadecenoic acid	Root	[27]

**Table 4:** Terpenoids from *Cortex Periplocae* (77–88).

No.	Compound’s Name	Parts Used	Reference
77	α-amyrinacetate	Root	[32]
78	β-amyrinacetate	Root	[33]
79	α-amyrin	Root	[34]
80	β-amyrin	Root	[35]
81	Lupeol acetate	Root	[32]
82	Ursolicacid	Root	[31]
83	Oleanolicacid	Root	[31]
84	Hederagenin	Root	[36]
85	Lupeol	Root	[37]
86	Cycloeucalenol	Root	[38]
87	(24*S*)-9,19-cycloart-25-ene-3β,24-diol	Root	[39]
88	(24*R*)-9,19-cycloart-25-ene-3β,24-diol	Root	[39]

**Table 5:** Others components from *Cortex Periplocae* (89–108).

No.	Compound’s Name	Parts Used	Reference
89	Perisaccharide A	Root	[1]
90	Perisaccharide B	Root	[1]
91	Perisaccharide C	Root	[1]
92	Oligosaccharides C_1_	Root	[40]
93	Oligosaccharides D_2_	Root	[40]
94	Oligosaccharides F_2_	Root	[40]
95	Oligosaccharides F_1_	Root	[40]
96	β-sitosterol	Root	[11]
97	Baohuoside I	Root	[41]
98	Daucosterol	Root	[42]
99	Tortoside B	Root	[43]
100	Limonin	Root	[44]
101	Proanthocyandin B_2_	Root	[45]
102	Ethyl caffeate	Root	[43]
103	Scopoletin	Root	[46]
104	4-Methoxybenzaldehyde	Root	[20]
105	4-*O*-(2-*O*-acetyl-β-d-digitalopyranosyl)-d-cymaropyranose	Root	[47]
106	Methyl-4-*O*-(2-*O*-acetyl-β-d-digitalopyranosyl)-β-d-cymaropyranoside	Root	[47]
107	Vanillin lactose glycolsides	Root	[33]
108	β-sitosterol-β-d-glucoside	Root	[11]

## Abbreviations

LD_50_	Lethal Dose 50%
PK	pharmacokinetics
Tmax	Cmax, maximum plasma concentration
IS	internal standard
LVSP	left ventricular systolic pressure
LVEDP	left ventricular end-diastolic pressure
